# Molecular Characterization of Novel Totivirus-Like Double-Stranded RNAs from *Puccinia striiformis* f. sp. *tritici*, the Causal Agent of Wheat Stripe Rust

**DOI:** 10.3389/fmicb.2017.01960

**Published:** 2017-10-10

**Authors:** Li Zheng, Xia Lu, Xiaofei Liang, Shuchang Jiang, Jing Zhao, Gangming Zhan, Peng Liu, Jianhui Wu, Zhensheng Kang

**Affiliations:** State Key Laboratory of Crop Stress Biology for Arid Areas and College of Plant Protection, Northwest A&F University, Yangling, China

**Keywords:** wheat stripe rust, *Puccinia striiformis*, deep sequencing, mycovirus, *Totivirus*

## Abstract

Characterization of newly isolated mycoviruses may contribute to understanding of the evolution and diversity of viruses. Here, a deep sequencing approach was used to analyze the double-stranded RNA (dsRNA) mycoviruses isolated from field-collected *P. striiformis* samples in China. Database searches showed the presence of at least four totivirus-like sequences, termed *Puccinia striiformis* virus 1 to 4 (PsV1 to 4). All of these identified sequences contained two overlapping open reading frames (ORFs) which encode a putative coat protein (CP) and an RNA-dependent RNA polymerase (RdRp) showing similar structures to members of the genus *Totivirus*. Each PsV contained a -1 ribosomal frameshifting region with a slippery site and a pseudoknot structure in the overlapped regions of these ORFs, indicating that the RdRp is translated as a CP-RdRp fusion. Phylogenetic analyses based on RdRp and CP suggested that these novel viruses belong to the genus *Totivirus* in the family *Totiviridae*. The presences of these PsVs were further validated by transmission electron microscope (TEM) and RT-PCR. Taken together, our results demonstrate the presence of diverse, novel totiviruses in the *P. striiformis* field populations.

## Introduction

Mycoviruses (fungal viruses) are of common occurrence in all major taxonomic groups of filamentous fungi, yeasts and oomycetes (Pearson et al., 2009; Ghabrial et al., 2015). Although most mycoviruses infect their fungal hosts symptomlessly (cryptic infections), some of them cause phenotypic alterations including hypovirulence and debilitation, and thus can be used as biological control agents against fungal diseases, such as the +ssRNA mycovirus Cryphonectria hypovirus 1 (CHV1) against *Cryphonectia parasitica* (Nuss, 2005) and the ssDNA mycovirus Sclerotinia sclerotiorum hypovirulence-associated DNA virus 1 (SsHADV-1) against *S. sclerotiorum* (Yu et al., 2013). Most mycoviruses have double-stranded RNA (dsRNA) genomes and form typical virus particles (Lin et al., 2012; Zheng et al., 2014). DsRNA mycoviruses are now classified into six families, including *Totiviridae*, *Partitiviridae*, *Megabirnaviridae*, *Reoviridae*, *Quadriviridae*, *Chrysoviridae* (Kondo et al., 2016). Among them, the families *Totiviridae* and *Partitiviridae* are the largest.

The Basidiomycota rust fungi cause disease on a variety of host crop species including soybean, coffee, groundnuts, wheat, and tree species such as conifers and poplars. Rust fungi belong to the order Pucciniales which consists of over 5,000 species and over 100 genera (Zhang et al., 1994). Rusts are obligate biotrophs, which can only absorb nutrients from alive host tissue. Wheat stripe rust, caused by *Puccinia striiformis* f. sp. *tritici*, is one of the most important diseases of wheat worldwide (Chen, 2005; Wellings, 2011). In China, the annual yield loss was estimated to be approximately 1.0 million metric tons (Chen et al., 2009). The presence of dsRNA in rusts was first reported in Newton et al. (1985). Subsequently, indirect evidence suggested the presence of mycovirus-like RNA molecules in *Phakopsora pachyrhizi*, the causal agent of Asian soybean rust (Link et al., 2014). Recently, a dsRNA mycovirus was identified from the fungus *P. pachyrhizi* (Cooper et al., 2016). Although some progress has been made in obtaining dsRNAs from rust mycoviruses, little is known about their genome organization, which is due in a large extent to their asymptomatic infections (Zhang et al., 1994). The cryptic dsRNAs are mostly reported in the families *Totiviridae* and *Partitiviridae* (Ghabrial, 1998; Zheng et al., 2014). The family *Totiviridae* currently comprises five approved genera, of which totiviruses and victoriviruses infect only fungi, while giardiaviruses, trichomonasviruses and leishmaniaviruses infect mainly protozoa (Goodman et al., 2011; Kondo et al., 2016). Members of this family have non-segmented dsRNA genomes being 4.6–7.0 kbp in length and usually contain two large, partially overlapping open reading frames (ORFs) which encode a capsid protein (CP) and an RNA-dependent RNA polymerase (RdRp) respectively (Ghabrial et al., 2015).

Here we report four new complete totivirus-like genome sequences based on deep sequencing of dsRNAs isolated from field-collected *P. striiformis* samples in China. Based on viral genome organization, phylogeny and particle morphology, these novel viruses were identified to belong to the genus *Totivirus* in the family *Totiviridae*.

## Materials and Methods

### *P. striiformis* Fungal Samples

*Puccinia striiformis* urediniospores were obtained from the susceptible wheat cultivar Mingxian 169 in an experimental field (approximately 3 m × 6 m area) at the Northwest A&F University, Yangling, Shaanxi, China in the summer of 2015. Samples were collected and stored in a desiccator at 4°C. The *Puccinia striiformis* species identity of these field samples were validated based on the complete internal transcribed spacer (ITS) sequence of ribosomal DNA (rDNA). Total genomic DNA was extracted from uredinospores of *P. striiformis* with CTAB method as described by Justesen et al. (2002). The ribosomal rDNA-ITS primers, ITS1 (5′-TCCGTAGGTGAACCTGCGG-3′) and ITS4 (5′-TCCTCCGCTTATTGATATGC-3′), were synthesized by Sangon Bio-Tech Co., Ltd. Polymerase chain reaction (PCR) amplification was done following standard methods according to Zheng et al. (2017).

### Extraction of dsRNA

DsRNA was extracted from 1.0 g of *P. striiformis* urediniospores according a described method with minor modifications (Zheng et al., 2014), and absorption column made up of cellulose powder CF-11 (Whatman, United Kingdom) was used. Extracted dsRNAs were treated with DNase I and S1 nuclease (TaKaRa Bio Inc) to remove genomic DNA and single-stranded RNA (ssRNA) contaminations, the qualities of which were then analyzed based on 1.0% (w/v) agarose gel electrophoresis.

### cDNA Library Construction and Illumina Sequencing

The dsRNA sample (1.0 μg) was used for cDNA library construction using the NEBNext^®^Ultra^TM^ RNA Library Prep Kit (Illumina, United States) following manufacturer’s instructions. The cDNA is end-repaired and adenylated prior to adaptor ligation, library construction and amplification under this method. Then the sequenced-ready library was subjected to 5 million of 150 nucleotide (nt) paired-end reads using Illumina HiSeq 4000 technology. The cDNA library construction and deep sequencing analysis were carried out by Shanghai Hanyu Bio-Tech Co., Ltd. After deep sequencing (>40,000 × coverage), raw reads were cleaned by removing adapter sequences and low-quality bases (PHRED quality scores ≤ 5), and truncated reads smaller than 35 bp were discard. 29 contigs were obtained and the N50 length is 1,489 nt. The total length of sequencing is about 37 kb with a GC content of 42.06%. *De novo* assembly of contiguous sequences was conducted using the Velvet de novo assembly algorithm with k-mer: 69. Minimum contig length was 500 bp as well as minimum coverage was 18. The ends and the other parts of the sequences were all confirmed by Sanger sequencing. To obtain the termini of the dsRNAs, rapid amplification of cDNA ends (RACE) was performed (Zheng et al., 2013).

### Database Search and Sequence Analysis

Open reading frames (ORFs) were identified using the National Center for Biotechnology Information (NCBI) ORF Finder program^1^. Motif searches were performed in three databases, including PROSITE^2^, Pfam^3^ and CDD^4^. RNA pseudoknot structure was predicted using the DotKnot program and drawn with PseudoViewer3 (Byun and Han, 2009; Sperschneider and Datta, 2010).

### Phylogenetic Analysis

Phylogenetic trees were constructed based on the deduced amino acid sequences of the putative RdRp and CP regions using the maximum-likelihood (ML) method of the MEGA program (version 6.0)with 1,000 bootstrap replicates as described previously with minor modifications (Kondo et al., 2016). Multiple alignments of the sequences of RdRp and CP were conducted with Clustal-X program (Thompson et al., 1997).

### Purification of Viral Particles and Electron Microscopy

Virus particles were purified using the method as described previously with minor modifications (Sanderlin and Ghabrial, 1978). Three gram *P. striiformis* urediniospores were grounded into fine powder in the presence of liquid nitrogen with a sterilized mortar and pestle. The powder was then mixed with 200 ml extraction buffer made up of 0.1 M sodium phosphate, and pH 7.0 containing 3% Triton X-100. The suspension was centrifuged at 10,000 × *g* for 20 min to remove the spore cell debris. Subsequently, the supernatant was subject to a 1.5 h ultracentrifugation at 120,000 × *g* for viral particle precipitation. The pellets were then suspended in 0.1 M sodium phosphate buffer and centrifuged at 15,000 × *g* for 30 min. Then the suspension containing the virus particles was fractionated via a 10–40% (w/v) sucrose gradient by centrifugation at 60,000 × *g* for 3.5 h. Fractions in the middle portion of the tube were collected, and stained with 2% phosphotungstic acid (pH 7.4) and observed under a transmission electron microscope (TEM) (HT7700, Japan).

### Validation of the Presence of Mycovirus-Like dsRNAs in Isolated Viral Particles

The dsRNAs from virus particles was extracted using phenol, chloroform and isoamyl alcohol, and then subjected to electrophoresis in 1% (w/v) agarose gel. Using the dsRNAs from virus particles as templates, complementary DNAs were synthesized as described by Xie et al. (2011) with tagged random dN6 primers (Rong et al., 2002; Zheng et al., 2013). Reverse transcription polymerase chain reaction (RT-PCR) was then performed with specific primers (Supplementary Table S1) which target the four PsVs respectively according to the method of Vainio et al. (2012) with minor modifications.

## Results

### *P. striiformis* Urediniospores Contain Putative Totiviruses-Like dsRNAs

**FIGURE 1:**
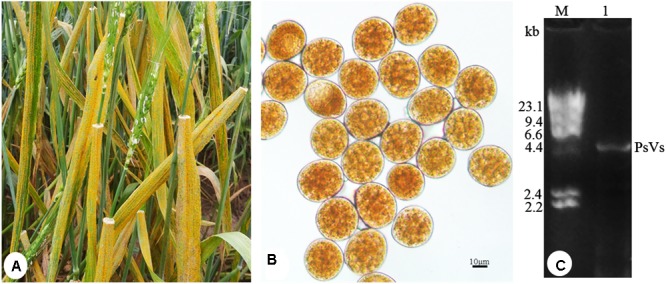
*Puccinia striiformis* f. sp. *tritici* urediniospores contain dsRNA-like nucleic acids elements. **(A)** Symptoms of stripe rust disease on wheat leaves. **(B)** *P. striiformis* urediniospores collected from wheat leaves. **(C)** Agarose gel electrophoresis of dsRNA extracted from the urediniospores of *P. striiformis*. M indicates molecular markers of aaaDNA digested with *Hind* III.

To detect dsRNA virus, *P. striiformis* urediniospores were collected from heavily infected wheat leaves growing in the field (**Figure 1A**). Urediniospores were round-shaped and approximately 30 μm in diameter (**Figure 1B**). The sequence length of ITS is 663 bp and it has 99% sequence identity to the *P. striiformis* strain CYR32. The result confirmed the fungus belonged to *P. striiformis* species. Nucleic acid extraction obtained nucleic acid bands of approximately 5.0 kb in size (**Figure 1C**) that resisted DNase I and S1 nuclease digestions, indicating the presence of dsRNA-like nucleotides.

Four *P. striiformis*-derived totivirus-like sequences, termed as Puccinia striiformis virus 1 to 4 (PsV1 to 4) (Supplementary Table S2), were identified from the post-assembly contigs by deep sequencing. The 5′- and 3′- untranslated regions (UTRs) of all PsVs were obtained. The complete nucleotide sequences of PsVs (1 to 4), ranging from 5,008 to 5,061 nt, were all deposited in GenBank under accession numbers KY207361-KY207364, respectively.

**FIGURE 2:**
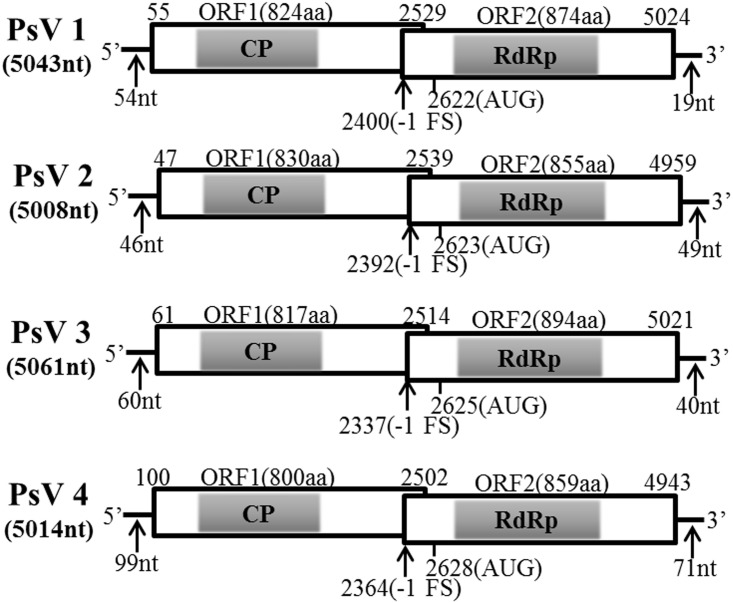
Genomic organizations of PsVs sequences. The two overlapping ORFs and the untranslated regions (UTRs) are shown by open boxes and a single line, respectively. The conserved CPs and RdRps domain are indicated by shadows. Nucleotide positions of ORFs and the putative slippery site for -1 frameshifting are shown with black arrows.

**FIGURE 3:**
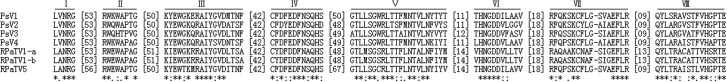
Sequence alignment of PsV RdRp motifs with those of selected viruses in the genus *Totivirus*. Horizontal lines above the alignment indicate the eight motifs, numbers in brackets suggest the amino acid positions, shadow area and asterisks indicate identical amino acid residues, colons indicate the similar residues.

Pair-wise comparisons among proteins encoded by PsV1 to 4 revealed moderate levels (37–42%) of amino acid sequence identity of RdRp, but low-level similarities (31–33%) among CP. It is notable that the 5′-end sequences of PsVs 1 to 4 (5′-AUAAAUCCCC…-3′, 5′-AUACAAUCCCC…-3′, 5′-AUAUAACUCCC…-3′ and 5′-AUAAAACCCCC…-3′, respectively) appeared to be partially conserved. Likewise, the 3′-end sequences were not conserved (data not shown). All four genomes contained two ORFs encoding CP and RdRp respectively (ORF1 and ORF2) (**Figure 2**), such organization is typical of totiviruses. The predicted CP and RdRp proteins showed moderate levels of amino acid sequence identities (32–34% for CP and 39–41% for RdRp, respectively) with Phakopsora pachyrhizi mycovirus (PpV) and red clover powdery mildew-associated totivirus 5 (RPaTV5) (Supplementary Table S3). A search of conserved domain database (CDD) and multiple protein alignment confirmed that the predicted RdRp domains contain eight conserved motifs (I to VIII), including the GDD motif, which are the typical characteristics of mycovirual RdRps (**Figure 3**) (Routhier and Bruenn, 1998).

**FIGURE 4:**
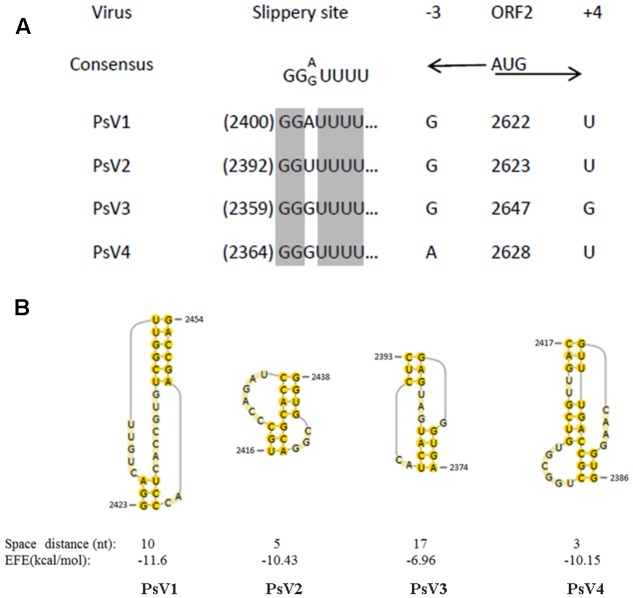
Putative slippery sites and pseudoknot structures predicted in PsVs. **(A)** The putative slippery sequences (XXXYYYZ) involved in -1 translational frameshift. The PsVs nucleotide sequences at –3 and +4 relative to the AUG start codon are indicated by black arrows. **(B)** The predicted pseudoknot structures downstream of the potential slippery sequences for –1 frameshifting are shown by yellow shadow. Spacer distance represents the number of nucleotides between the slippery site and the pseudoknot. EFE (kcal/mol) indicates the minimal free energy.

The PsV sequences between position -3 and +4 relative to the ORF2 AUG start codons (**Figure 4A**) are similar to the translation initiation sites of the viruses of plants and yeasts (Lütcke et al., 1987; Hamilton et al., 1987). Sequence analysis of PsVs indicated that there is an overlap region between ORFs 1 and 2 (**Figure 2**). It is therefore possible that ORFs 2 of PsVs is translated as a fusion protein with ORFs 1 through a -1 ribosomal frameshift which is a canonical slippery sites ‘XXXYYYZ’ (where X is A/C/G/U, Y is A/U, and Z is A/C/U) within the overlapping region (Bekaert and Rousset, 2005). The slippery site ‘GGA/GUUU’ sequence in PsV1 to 4 sequences (**Figure 4B**) is similar to these of the RPaTVs (Kondo et al., 2016). Using the DotKnot program, a pseudoknot structure was predicted in the downstream of each putative slippery site (**Figure 4B**). RNA pseudoknots are known to help pausing the translating ribosome and increasing the frequency of frameshifting (Plant et al., 2003; Zhai et al., 2008; Kondo et al., 2016).

### Phylogenetic Analyses

**FIGURE 5:**
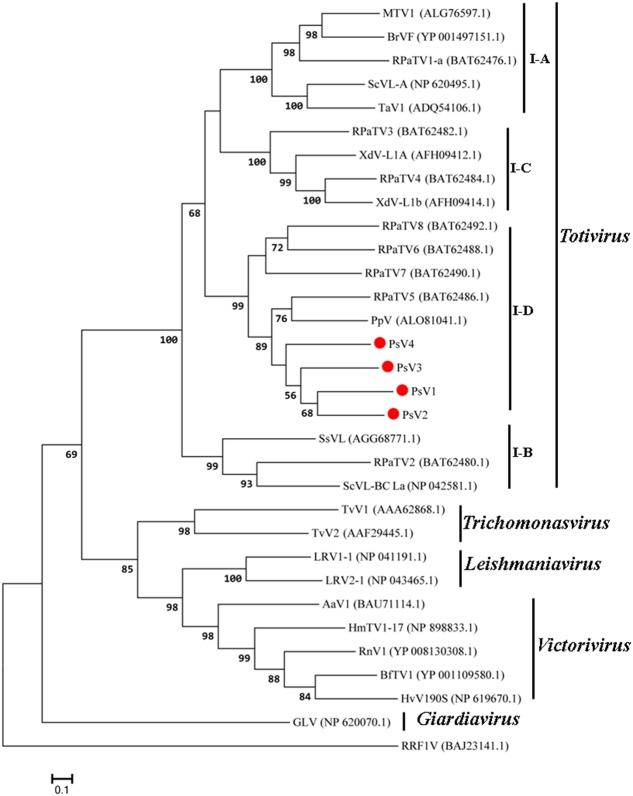
Phylogenetic analysis based on the deduced amino acid sequences of putative RdRps using the maximum likelihood (ML) method with 1,000 bootstrap replicates. The scale bar represents a genetic distance of 0.1 amino acid substitutions per site. Red circles indicate the novel mycoviruses PsVs in the present study.

**FIGURE 6:**
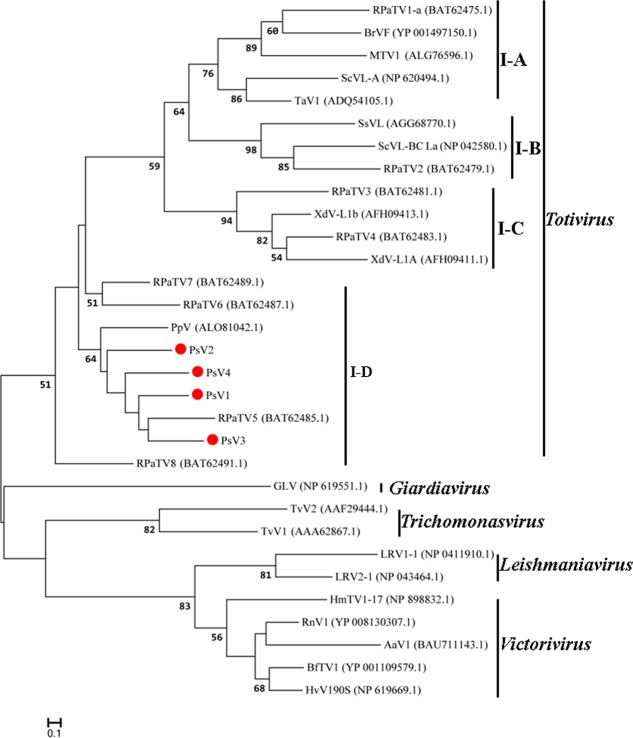
Phylogenetic analysis based on the deduced amino acid sequences of putative CPs using the maximum likelihood (ML) method with 1,000 bootstrap replicates. The scale bar represents a genetic distance of 0.1 amino acid substitutions per site. Red circles indicate the novel mycoviruses PsVs in the present study.

The maximum likelihood (ML) tree for RdRP is shown in **Figure 5**, *Totivirus* contained four subgroups, I-A, I-B, I-C and I-D. PsV1 to 4 clustered with RPaTVs 5 to 8 and PpV in the subgroup I-D. The ML phylogeny based on CPs is shown in **Figure 6**, which had similar topology as the one based on RdRPs.

Taken together, genome organizations, amino acid sequence alignments and phylogenetic analyses all support that PsV1 to 4 are new members of the genus *Totivirus* within the family *Totiviridae*.

### Observation of Viral Particle and Validation of the Viral Genome Sequences

**FIGURE 7:**
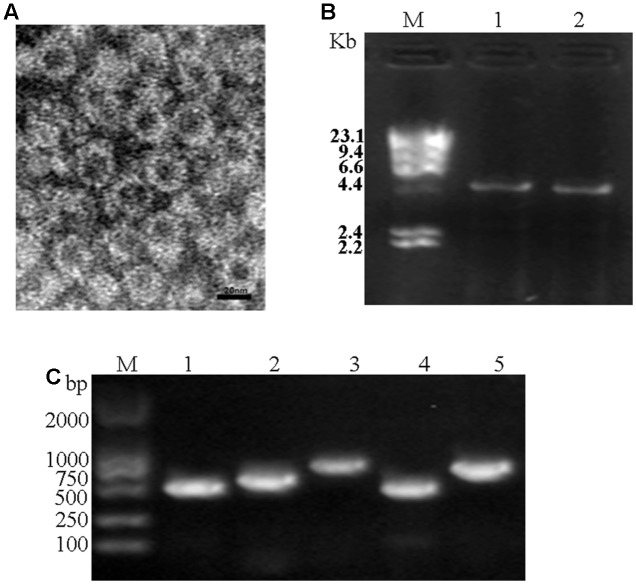
Electron microscopy and RT-PCR. **(A)** Viral particles purified from field-collected *P. striiformis* urediniospores. **(B)** Agarose gel electrophoresis of dsRNAs extracted from purified virus particles of PsVs (lane 1) and the urediospores of *P. striiformis* from field-collected samples (lane 2). **(C)** Confirmation of identity of dsRNAs extracted from purified virus particles using PsVs 1-4 specific primers (lanes 1-4, respectively) (Supplementary Table S1). M indicates molecular markers of aaaDNA digested with *Hind* III.

Under TEM, the viral particles purified from *P. striiformis* urediniospores were isometric with an average diameter of 35 nm (**Figure 7A**). The sizes of dsRNAs from viral particles were similar with the dsRNAs extracted directly from *P. striiformis* urediniospores (**Figure 7B**). Using the dsRNAs extracted from viral particles as templates, we performed RT-PCR based on PsV-specific primers targeting RdRps, which successfully amplified products and in all cases the sizes were identical to ones expected based on the PsV genomes (**Figure 7C**).

## Discussion

Characterization of newly isolated fungal viruses may contribute to understanding of the evolution and diversity of viruses (Chiba et al., 2009; Nibert et al., 2013; Zheng et al., 2014). Although mycoviruses have been identified from all major groups of filamentous fungi (Ghabrial and Suzuki, 2009), it is rarely reported in obligate biotrophs, such as rust fungi and powdery mildews most likely due to their unculturablity (Kondo et al., 2016). Recently, with the development of next generation sequencing technologies, new mycoviruses have been identified directly from field-collected fungal samples (Feldman et al., 2012; Kondo et al., 2016; Marzano and Domier, 2016). The present study revealed four complete (PsV1 to 4) totiviral sequences. The presence of these PsVs was further validated by TEM and RT-PCR. To the best of our knowledge, this study provides the first evidence of dsRNA mycoviruses infections in the *P. striiformis* f. sp. *tritici*, the causal agent of wheat stripe rust.

Each of the four PsV genomes contained two overlapping ORFs which encode the conserved domains of CP and RdRp, respectively. Moreover, the four PsVs were deduced to universally contain the -1 ribosomal frameshifting at the overlapping regions (**Figure 2**), which could facilitate translation of ORF1 and ORF2 as a fusion polyprotein. The predicted ORF2 coding strategy of PsVs were in line with members of genus *Totivirus* in the family *Totiviridae*, such as Saccharomyces cerevisiae virus L-A (ScVL-A) (Dinman et al., 1991) and red clover powdery mildew-associated totiviruses (RPaTVs) (Kondo et al., 2016).

Phylogenetic analysis with RdRp and CP sequences placed PsVs in a distinctive clade with members of *Totivirus* in the family *Totiviridae* (**Figures 5**, **6**). Interestingly, both RdRp and CP phylogeny placed PsV1 to 4 together with RPaTVs 5 to 8 and PpV in the subgroup I-D. Interestingly, all the virus hosts in subgroup I-D are obligate biotrophic fungi, such as *P. striiformis*, *P. pachyrhizi*, and powdery mildew fungi, indicating an unknown interaction between obligate biotrophic fungi and totiviruses in this subgroup.

The current criteria for species demarcations of the genus *Totivirus* require less than 50% sequence identity of CP/RdRp proteins (Wickner et al., 2011). In the present study, proteins encoded by PsV1 to 4 shared moderate levels (RdRp, 37–42%; CP, 31–33%) of amino acid sequence identities to known totivirus species and among themselves, based on which they should represent novel totivirus species.

Taken together, this study characterized the molecular features of PsVs present in field-collected samples of wheat stripe rust fungus. The four novel viruses PsVs belong to the genus *Totivirus* in the family *Totiviridae.* This study demonstrates the presence of diverse, novel totiviruses in the *P. striiformis* f. sp. *tritici* populations, characterizing their interactions with the *P. striiformis* host will potentially allow for developing novel rust disease control strategies.

## Author Contributions

ZK designed experiments; LZ performed the experiments; LZ, XL, XfL, and SJ analyzed the data; JZ, GZ, PL, and JW joined the discussion and gave the original ideas; LZ wrote the paper.

## Conflict of Interest Statement

The authors declare that the research was conducted in the absence of any commercial or financial relationships that could be construed as a potential conflict of interest.

## Footnotes

1 http://www.bioinformatics.org/sms2/orf_find.html

2 http://www.expasy.ch/

3 http://pfam.sanger.ac.uk/

4 http://www.ncbi.nlm.nih.gov/Structure/cdd/wrpsb.cgi
