# Supplementary material for: Molecular Characterization of Novel Totivirus-Like Double-Stranded RNAs from Puccinia striiformis f. sp. tritici, the Causal Agent of Wheat Stripe Rust
Source: Front Microbiol. 2017 Oct 10;8:1960. doi: 10.3389/fmicb.2017.01960 (PMC5641321; doi:10.3389/fmicb.2017.01960)
Supplement: Supplementary file 1 [file Presentation_1.PDF]

Supplementary Material

Molecular characterization of novel totivirus-like double-stranded RNAs from *Puccinia striiformis* f. sp. *tritici*, the causal agent of wheat stripe rust

First Author*, Li Zheng

*** Correspondence: Zhensheng Kang;** kangzs@nwsuaf.edu.cn

1. **Supplementary Tables**

TABLE S1. Species-specific PsVs primers used for RT-PCR detection

PsV species	Primer name	Sequence 5'-3'	Amplicon size
PsV1	PsV1-F	GCAAACAACGGTCAGAGT	544 bp
	PsV1-R	AAAGTCTAATACCGATGA	
PsV2	PsV2-F	CTTGACGGGTTCAGGACA	657 bp
	PsV2-R	AACCAACACTTCCAACTC	
PsV3	PsV3-F	CAGGATAGGTGAAGGTGGT	743 bp
	PsV3-R	CTTCTGCTGCTACTTTGT	

PsV4	PsV4-F	TAAACTACCCACCTGACA	557 bp
	PsV4-R	TACGGTATTTGCGTCTAT	

TABLE S2. Four putative totivirus-like sequences from the field-collected samples of *P. striiformis* f. sp. *tritici*.

Virus name	Abbreviation	Size (nt)	SG [a]	Accession number
Puccinia striiformis totivirus 1	PsV1	5043	I-D	KY207361
Puccinia striiformis totivirus2	PsV2	5008	I-D	KY207362
Puccinia striiformis totivirus 3	PsV3	5061	I-D	KY207363
Puccinia striiformis totivirus 4	PsV4	5014	I-D	KY207364

[a] Subgrouping based on the RdRp or CP phylogenetic tree (Figure 5, 6).

TABLE S3. Summary of the BLASTp search results for PsVs.

Virus name	Size (aa, kDa)	Best-matched virus	Identity(e-value, QC[b])
**QS[a]: ORF1 (CP)**			
PsV1	824, 94	Phakopsora pachyrhizi mycovirus	34% (2e^{-159},97)
PsV2	830, 94	Phakopsora pachyrhizi mycovirus	33% (2e^{-151}, 96)
PsV3	817, 92	Phakopsora pachyrhizi mycovirus	32% (2e^{-129}, 88)
PsV4	800, 90	Phakopsora pachyrhizi mycovirus	35% (1e^{-135}, 88)
**QS: ORF2 (RdRp)**			
PsV1	874, 99	Red clover powdery mildew-associated totivirus 5	41% (0.0, 92)
PsV2	855, 97	Red clover powdery mildew-associated totivirus 5	39% (0.0, 94)
PsV3	894, 101	Red clover powdery mildew-associated totivirus 5	39% (0.0, 92)
PsV4	859, 98	Red clover powdery mildew-associated totivirus 5	39% (0.0, 91)

[a] QS : query sequence.

[b] QC: query coverage (%).
